# P-1020. Real-World Effectiveness and Health-Related Quality of Life Improvements Using Fecal Microbiota, Live-jslm for the Prevention of Recurrent Clostridioides difficile Infection

**DOI:** 10.1093/ofid/ofaf695.1216

**Published:** 2026-01-11

**Authors:** Richard L Hengel, Sujatha Krishnan, Timothy E Ritter, Jonathan A Rosenberg, Kathy A Baker, Lucinda J Van Anglen, Kelly E Hanna, Mielad Moosapanah, Sanghyuk Seo, Kevin W Garey

**Affiliations:** Atlanta ID Group, Atlanta, GA; DFW Infectious Diseases, PLLC, Frisco, Texas; GI Alliance, Southlake, Texas; GI Alliance, Southlake, Texas; Texas Christian University, Fort Worth, Texas; Healix Infusion Therapy, LLC, Sugar Land, Texas; Healix Infusion Therapy, LLC, Sugar Land, Texas; Ferring Pharmaceuticals, Inc., Parsippany, New Jersey; Ferring Pharmaceuticals, Inc., Parsippany, New Jersey; University of Houston, Houston, Texas

## Abstract

**Background:**

Fecal microbiota, live-jslm (RBL) is a microbiota-based product approved for the prevention of recurrence of *Clostridioides difficile* infection (rCDI) in adults following antibiotic treatment and was demonstrated to be safe and efficacious in clinical trials. Recurrence of CDI is frequent and has been shown to negatively impact health-related quality of life (HR-QOL). This study reports the effectiveness of RBL and assessment of HR-QOL in an outpatient real-world setting.

**Table 1.**
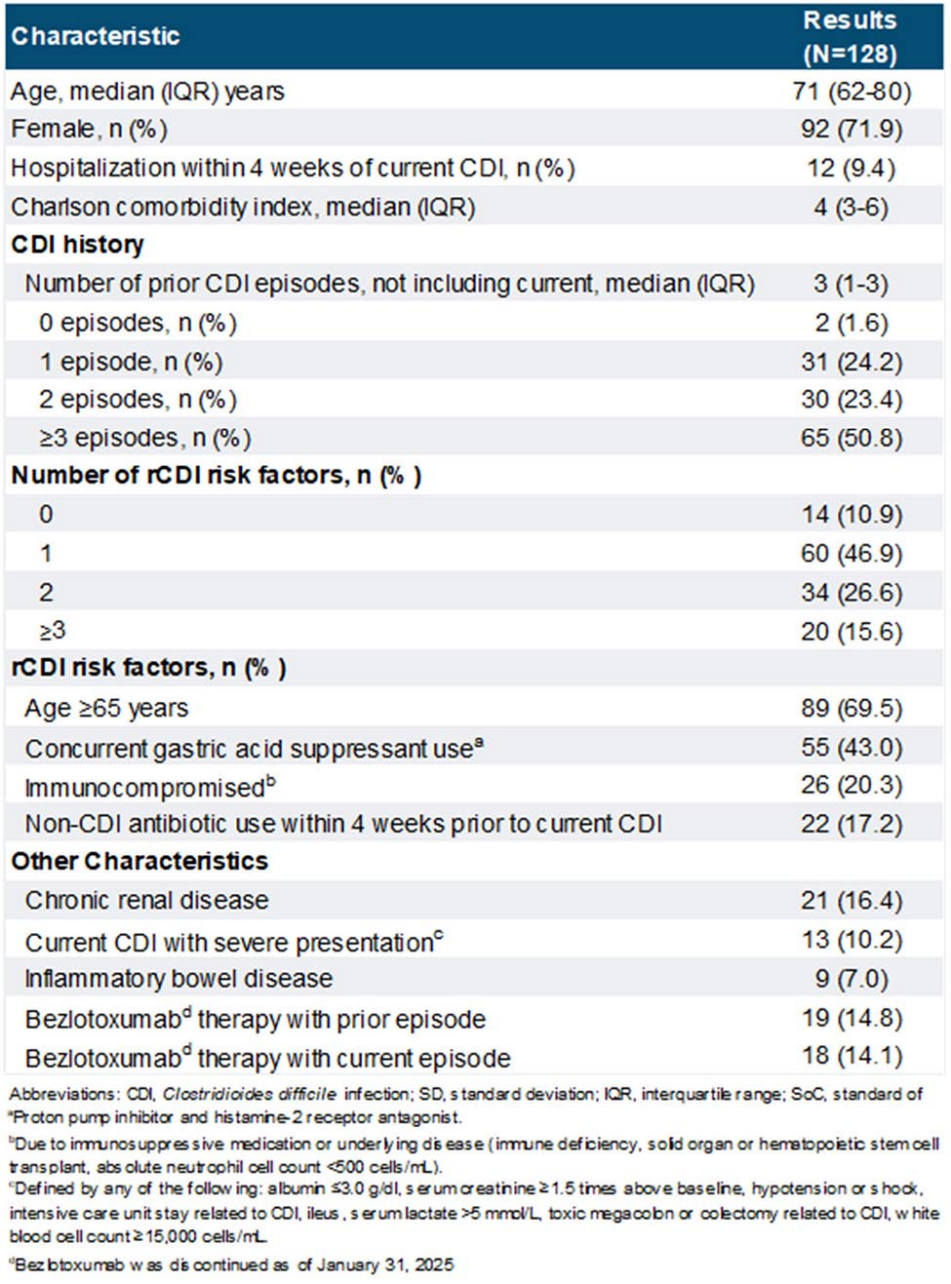
Study Cohort Demographics and Treatment Characteristics

**Figure 1.**
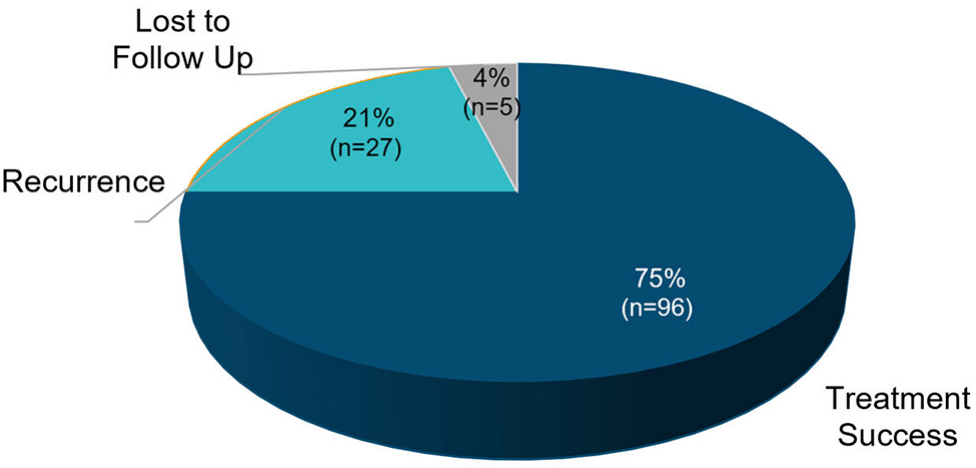
Treatment Outcomes (N=128)

**Methods:**

This single-arm, multicenter study included patients ≥18 years with rCDI who received RBL in physician offices from February 2023 to March 2025. Data included patient demographics, comorbidities, rCDI history, risk factors, RBL therapy characteristics, standard of care antibiotic (SoC), other related treatments and RBL-related adverse events. Recurrence, defined as 3 or more occurrences of diarrhea within 24 hours, was assessed at 8 weeks post-RBL administration. HR-QOL was assessed at baseline and at 8 weeks with the Cdiff32, a validated, disease-specific patient-reported instrument to evaluate changes in HR-QOL associated with CDI.

**Figure 2.**
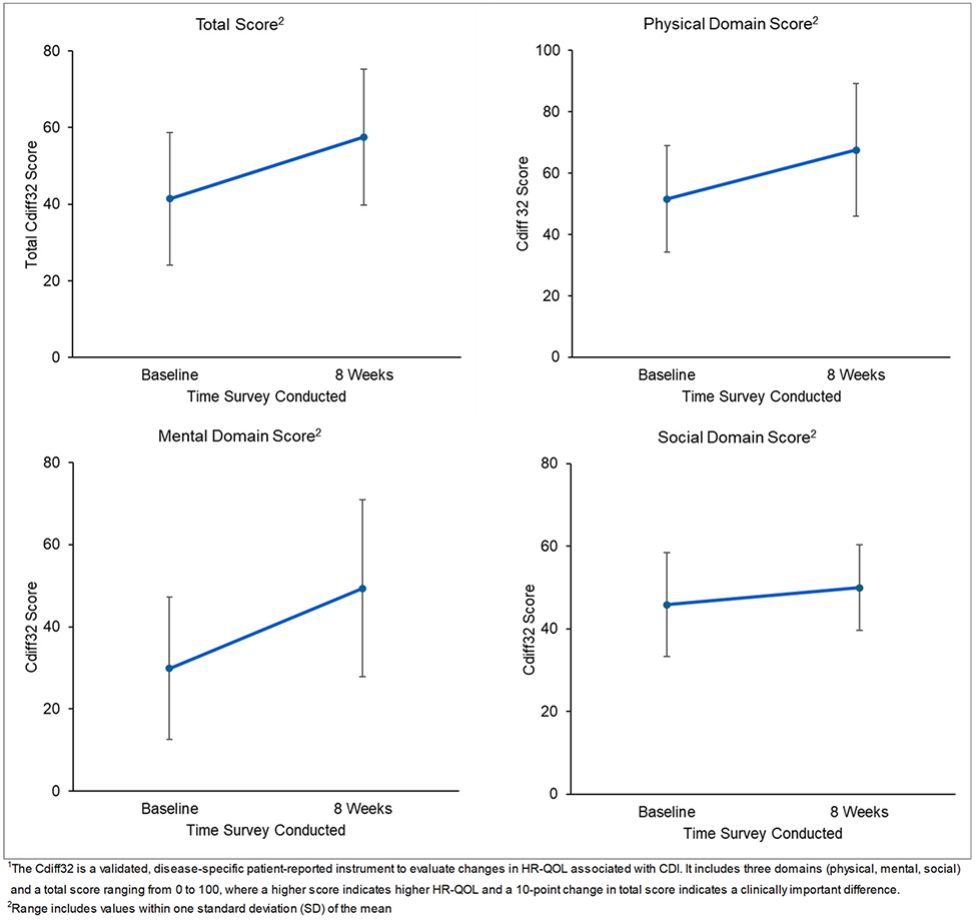
Health-Related Quality of Life over Time with the Cdiff32 Survey1 (n=40)

**Results:**

RBL was administered to 128 patients. Patient characteristics are shown in Table 1. Patients had a median age of 71 (IQR: 62-80) years and were predominantly female (71.9%). Patients had multiple comorbidities (Charlson score 4; IQR: 3-6) and 42.2% had 2 or more rCDI risk factors. The most common risk factors were age ≥65 years (69.5%) and concurrent gastric acid suppressant use (43.0%). The median number of prior CDI episodes was 3 (IQR: 1-3). Overall, 75% had treatment success at 8 weeks, 21% had a recurrence and 4% were lost to follow-up (Figure 1). A total of 40 patients completed both baseline and 8-week HR-QOL surveys. Mean±SD HR-QOL improved from baseline (41.4±17.3) to week 8 (57.5±17.8). Improvements in scores were seen in all domains from baseline to week 8 (Figure 2).

**Conclusion:**

This real-world study demonstrated that RBL treatment to prevent recurrence of CDI was safe and effective in routine clinical practice, with clinically meaningful improvements observed in HR-QOL between baseline and 8-weeks.

**Disclosures:**

Richard L. Hengel, MD, FIDSA, Gilead: Grant/Research Support Timothy E. Ritter, MD, Abbvie: Advisor/Consultant|Boehringer Ingelheim: Advisor/Consultant|Bristol Myers Squibb/Celgene: Advisor/Consultant|Eli Lilly: Advisor/Consultant|Ferring: Advisor/Consultant|Genentech/Roche: Advisor/Consultant|Gilead: Advisor/Consultant|Intercept: Advisor/Consultant|Iterative Health: Advisor/Consultant|Janssen: Advisor/Consultant|Merck: Advisor/Consultant|Pfizer: Advisor/Consultant|Sanofi: Advisor/Consultant|Spyre Therapeutics: Advisor/Consultant|Takeda: Advisor/Consultant Jonathan A. Rosenberg, MD, Aimmune: Advisor/Consultant|Ferring Pharmaceuticals: Advisor/Consultant Lucinda J. Van Anglen, PharmD, FIDSA, Cumberland Pharmaceuticals: Grant/Research Support|Ferring Pharmaceuticals: Grant/Research Support|Melinta Therapeutics: Grant/Research Support|Novartis: Grant/Research Support|Takeda Pharmaceuticals: Grant/Research Support Mielad Moosapanah, PharmD, Ferring Pharmaceuticals: Employee Sanghyuk Seo, PharmD, Ferring Pharmaceuticals: Employee Kevin W. Garey, PharmD, MS, FIDSA, FASHP, Acurx: Grant/Research Support|Merck & Co.: Grant/Research Support|Paratek Pharmaceuticals: Grant/Research Support

